# Hydration affects the physical and mechanical properties of baleen tissue

**DOI:** 10.1098/rsos.160591

**Published:** 2016-10-26

**Authors:** Alexander J. Werth, Robert W. Harriss, Michael V. Rosario, J. Craig George, Todd L. Sformo

**Affiliations:** 1 Department of Biology, Hampden-Sydney College, Hampden-Sydney, VA 23943, USA; 2 Department of Biology, Duke University, Durham, NC 27706, USA; 3 Department of Wildlife Management, North Slope Borough, Barrow, AK 99723, USA

**Keywords:** mysticete, whale, stiffness, histology, biomechanics, hydrophilic

## Abstract

Baleen, an anisotropic oral filtering tissue found only in the mouth of mysticete whales and made solely of alpha-keratin, exhibits markedly differing physical and mechanical properties between dried or (as in life) hydrated states. On average baleen is 32.35% water by weight in North Atlantic right whales (*Eubalaena glacialis*) and 34.37% in bowhead whales (*Balaena mysticetus*). Baleen's wettability measured by water droplet contact angles shows that dried baleen is hydrophobic whereas hydrated baleen is highly hydrophilic. Three-point flexural bending tests of mechanical strength reveal that baleen is strong yet ductile. Dried baleen is brittle and shatters at about 20–30 N mm^−2^ but hydrated baleen is less stiff; it bends with little force and absorbed water is squeezed out when force is applied. Maximum recorded stress was 4× higher in dried (mean 14.29 N mm^−2^) versus hydrated (mean 3.69 N mm^−2^) baleen, and the flexural stiffness was >10× higher in dried (mean 633N mm^−2^) versus hydrated (mean 58 N mm^−2^) baleen. In addition to documenting hydration's powerful effects on baleen, this study indicates that baleen is far more pliant and malleable than commonly supposed, with implications for studies of baleen's structure and function as well as its susceptibility to oil or other hydrophobic pollutants.

## 1. Background

Baleen is a uniquely specialized oral tissue with no functional analogue or evolutionary homologue. It hangs in sheets (called plates or laminae) from the palate of whales, where it filters aggregations of small planktonic or nektonic prey from seawater. Baleen's origin approximately 30 Ma in the Oligocene Epoch was a key innovation that led to an adaptive radiation in the mammalian Suborder Mysticeti and the evolution of gigantic body size in whale species [1–3].

Baleen grows from palatal gum tissue as transversely arrayed, serially suspended triangular plates that form a bilateral pair of comb-like racks, with 150–350 plates per rack [4–7]. Baleen is made entirely of dead, cornified cells and fibres. It is wholly composed of alpha-keratin, a fibrous protein creating intermediate filaments [8,9] found in many mammalian integumentary structures including hair, horn, nails, claws and hooves [10,11], yet baleen grows throughout life from living keratinocyte cells in the gums, in the same manner and speed as other keratinous integumentary structures [12]. Each baleen plate (lamina) forms as a layered sandwich, with flattened cortical sheets of keratin (similar to nails) enclosing an inner medullary layer of cylindrical horn tubes of keratin (similar to hair) plus a diffuse matrix of intertubular keratinous horn cells [4,13]. Although baleen plates grow throughout life, they maintain a constant length (25–300+ cm, depending on species) because the most distal portions erode from mechanical and hydrodynamic wear during feeding [14]. As plates erode, the enclosed horn tubes emerge as free fringes, also commonly called baleen bristles, filaments, fibres or hairs [15]. The plates and fringes together alter water flow around and within the mouth to trap aggregations of prey [5,16–18].

Baleen is a dynamic tissue that must withstand remarkable forces and function over a whale's lifetime (greater than equal to 100 years) without clogging or breaking down [19]. Depending on the species of whale and its characteristic foraging method, baleen encounters hydrodynamic loads ranging from slow, steady-state continuous flow of 70–85 cm s^−1^ in right and bowhead whales [16,18,20,21] to explosive, intermittent jets of vast volumes of water (up to 100 000 l) expelled at an estimated 800–1000 kPa or more with approximately 106 N m^−2^ of force in the groove-throated or rorqual species such as blue, fin and humpback whales [19]. For baleen to function properly given this extraordinary loading regime, it must balance contradictory mechanical behaviours. Baleen must be both a hard, stiffened tissue that resists shear and other forces, yet at the same time a supple, flexible, tissue. If too stiff it will probably break; if too pliant it will probably bend too much from mechanical demands of repeated filtration. In either case, it is unlikely that the whale will capture sufficient prey to meet its metabolic needs.

How do baleen's structure and function—and especially the competing mechanical demands of stiffness and compliance—relate to its constant presence in water? Previous studies have investigated the general effects of water on keratin [22–24] or keratinous integumentary tissues [25], and specifically, the influence of hydration on keratin's mechanical properties [26,27]. Related studies have documented the effects of hydration on bone [28] and on chitin in arthropod cuticle [29,30]. However, the specific extent to which baleen tissue hydrates has not previously been investigated; the potential effects of water in determining or altering baleen's mechanical behaviour are unknown.

Remarkably, despite the fact that baleen is always submerged and hence fully hydrated *in vivo*, virtually all of what we know about baleen's form (at gross and microscopic levels) and function comes solely from dried specimens. Limited studies of baleen's biomechanical properties have been published [13,19,31] and these have not reported the basic role of hydration in this tissue's natural state. This is an essential question and the rationale for this study, given baleen's highly dynamic role in a fully aqueous environment. We predict that baleen's interaction with water affects its mechanical properties, and specifically that hydration is a major determinant of baleen's flexibility.

## 2. Material and methods

Full left and right racks of baleen were obtained from a juvenile North Atlantic right whale (*Eubalaena glacialis*, field number EgNEFL 1235) that died naturally after stranding on 18 December 2012, 2.8 miles north of Palm Coast, Florida, USA. All baleen was stored in a freezer in Jacksonville, Florida, for eight months. Three full plates (laminae) were removed, thawed, and shipped in plastic wrapping to Virginia. From these three baleen plates fifty 3 × 3 cm square samples were cut using a band saw: 40 square samples for mechanical testing and 10 samples for analysis of baleen's physical properties related to hydration. Thickness of the tissue squares was almost perfectly uniform, ranging from 2.82 to 2.95 mm (*n* = 50, s.d. = 0.03). Of the 10 samples for water content analysis, half of these samples were placed for 21 days in 12°C artificial seawater that was circulated to avoid surface biofilm formation or tissue degradation; the remaining five samples were allowed to dry uncovered in air (22–23°C with approximately 30% humidity). Before, during, and after this three week period the wet and dry samples were weighed every 3 days to determine water content, and following the 21 days the wet samples were allowed to air dry, whereas the dry samples were placed in flowing water, with weights again periodically measured over a second three week (21 day) period. In this way, the wet versus dry weights were analysed by determining how much water the initially dried baleen eventually gained and conversely how much weight the originally wet baleen lost after drying. Three additional control materials were tested in the experiments of hydration effects, also with five 3 × 3 cm squares of 3 mm thickness: aluminium, high density polyethylene (HDPE) plastic and wood (lauan plywood). All control samples had untreated surfaces. All wet samples of baleen and control materials were lightly patted to remove visible water drops prior to weighing.

**Figure 1.**
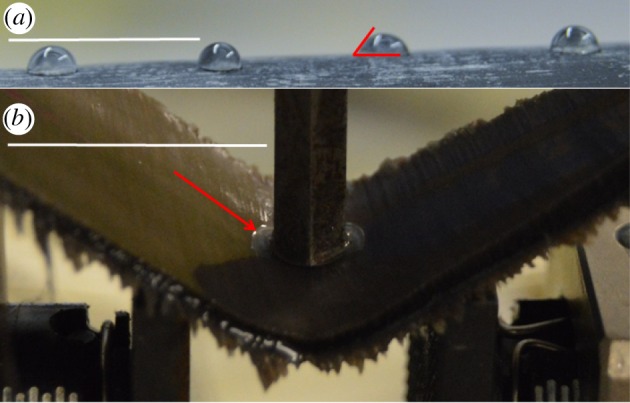
Wettability (*a*) was quantified by measuring contact angle on water droplets (shown here on dried baleen). Actual contact angles were measured on highly magnified images with the whole droplet not always fully visible. Hydrated baleen (*b*) is capable of bending without breaking and exudes water (red arrow) during mechanical strength testing. White scale bar in each image, 1 cm.

Following the full 42 days of weight analysis, testing of wettability (defined as adhesion of dissolved aqueous substance) was performed by determining the water's contact angle by placing micropipetted droplets of 5 µl of deionized water on both dried and hydrated samples of baleen. For each trial, 40 droplets were placed on the wet or dry baleen and immediately photographed laterally (figure 1*a*) using the direct static sessile drop method and optical analysis [32]. The photographs were examined using MB-Ruler 5.3 (Markus Bader, Berlin, Germany) to measure and record the precise angles at which each droplet sits on the baleen. Again, three control substances (aluminium, HDPE plastic, lauan plywood) were also tested via the same wet/dry contact angle technique.

The 40 square samples of baleen that had been set aside for mechanical strength testing were, like the pieces used for analysis of baleen's hydration properties, divided into two groups, half of which were kept in air and half of which were placed in flowing seawater at 12°C. The samples were kept and transported in air or water until moments prior to the testing on an Instron E1000 ElectroPuls universal testing machine (Instron/ITW Corp., Norwood, Massachusetts, USA) or Mark-10 ES30 universal testing machine with M4–200 force gauge (Mark-10 Corp., Copiague, New York, USA). To investigate the tissue's compressive strength via a simple three-point bending test, the small wet or dry baleen samples were placed on two arms spaced slightly apart: 18.92 mm for Instron testing and 24 mm for Mark-10 testing (figure 1*b*). The materials testing device recorded the force and resulting displacement, including the maximal force encountered by each tissue sample before failure or before the machine reached its limits, which occurred often with hydrated samples (figure 1*b*). Using the thickness and other dimensions of the square samples, and the distance between the points on which the samples lay, the flexural stress and strain were computed for each trial. From these values, the flexural stiffness (a function of the modulus) could also be determined for each trial. Because the stress–strain curves were not linear, flexural stiffness was defined as the largest slope regressed using a continuous subset with size 25% of the original data [33]. All initial baleen tests were performed with the Instron machine. These were repeated (with 40 new baleen samples) with the Mark-10 testing machine, and all tests on the three control materials (aluminium, HDPE plastic, lauan plywood) were also performed with the Mark-10 machine. Because our analysis uses a baleen dataset with pooled data from both machines, statistical tests were performed to compare results from the Instron versus Mark-10 testing. The same slope-finding algorithm [33] was used to determine flexural stiffness on data obtained from both machines.

After the initial results were analysed, more mechanical tests were performed on 30 additional 3 × 3 cm square samples, using baleen from the same original North Atlantic right whale. All samples were again stored for at least three weeks under the same conditions (in flowing artificial seawater at 12°C), but this time samples were tested (in three-point flexural bending on the Mark-10 machine) not only immediately but also at 15 min intervals following removal from water, until two hours had elapsed.

Supplementary field tests of baleen's water content were performed on four additional baleen plates from four bowhead whales (*Balaena mysticetus*) harvested by Inupiat subsistence hunters near Barrow, Alaska, in spring 2016. One small anterior plate was taken from near the tip of the rostrum in each whale: a 27.0 cm plate from whale 16B4, 34.5 cm from 16B6, 37.9 cm from 16B9, and 29.1 cm from 16B11. Each plate was placed in a sealed, moistened (with seawater) bag within 20 min of the whale being hauled out on the ice, weighed when back at the laboratory, and then dehydrated at 60°C for 1000 h (six weeks) to constant weight. Baleen samples were then rehydrated with seawater at room temperature (approx. 20°C) for 290 h (12 days) to constant weight for comparison of original and new weights.

## 3. Results

Tests of alternate drying and hydration indicate that right whale baleen is nearly one-third water by weight. The initially dried samples gained an average of 31.8% weight after three weeks' submersion in water (*n* = 5, $\bar{x}=1.57\;\text{g}$ gained, s.d. = 0.014), whereas the initially hydrated samples lost an average of 32.9% of their weight following three weeks in air (*n* = 5, $\bar{x}=1.31\;\text{g}$ lost, s.d. = 0.011). The overall mean water weight of all baleen samples was 32.28%. Tests of control materials showed no gain or loss of water for the aluminium and plastic samples, and 18.87% water weight for wood (*n* = 5, $\bar{x}=0.94\;\text{g}$ gained, s.d. = 1.46).

Tests of the supplementary field samples from bowhead whales showed that baleen of this species is 34.37% water (*n* = 4, s.d. = 2.2), with water content (g H_2_O g^−1^ dry mass) of mean 0.47 ± 0.04 (s.d.). Following 290 h of re-submergence, these samples gained back nearly all of their lost water weight, ending with mean 0.97% (±0.01 s.d.) of their original weight.

**Figure 2.**
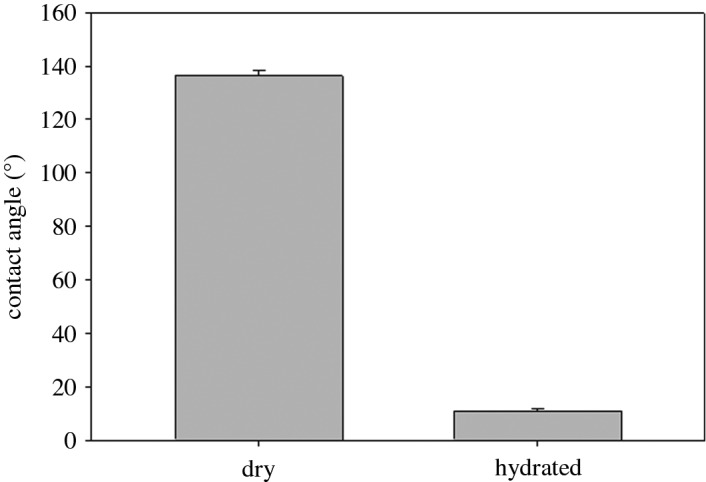
The mean contact angle (shown ± 1 s.d.) for dried baleen was obtuse (136.2°), indicating a hydrophobic material, whereas the contact angle for hydrated baleen was highly acute (11°) indicating instead an extremely hydrophilic material.

Wettability tests based on standard photography of contact angles with micropipetted water droplets (figure 1*a*) indicated a mean contact angle for dried right whale baleen of 136.2° (*n* = 40, s.d. = 12.49; figure 2), whereas hydrated samples had a mean contact angle of 11.0° (*n* = 18, s.d. = 0.61), showing a statistically significant difference (*p* = 0.02) via a *t*-test. Only 18 of the 40 attempts to measure the contact angle on the wet baleen samples were successful; in the other 27 trials, the 5 µl water droplet immediately collapsed and disappeared into the surface of the hydrated baleen sample. This occurred within 0.05–0.1 s, so that even videotaped trials that were analysed frame-by-frame could not provide conclusive contact angles. Generally contact angles of more than 90° indicate a highly hydrophobic material, with poor wettability and poor adhesion of any dissolved aqueous substance. Conversely, lower angles (less than 90°) reveal high wettability and good water adhesion, indicative of a hydrophilic material. The contact angles for the three control materials were as follows: aluminium 139° (*n* = 5, s.d. = 5.61), HDPE plastic 116° (*n* = 5, s.d. = 2.77) and plywood 38° (*n* = 5, s.d. = 4.39).

In terms of other general physical properties, wet baleen is darker in colour and shinier in appearance, with a slicker, smoother surface texture than dried baleen. Initial microscopic analysis [14] indicates a scuffed, scale-like surface in dried baleen, as tiny, cuticle-like keratin plates form a rough, ridged exterior. Pfeiffer [34] noted this in baleen fringes, but it has not yet been thoroughly described in a survey of baleen plates. Aside from darkening of the plywood, the control samples showed no changes in physical properties.

**Figure 3.**
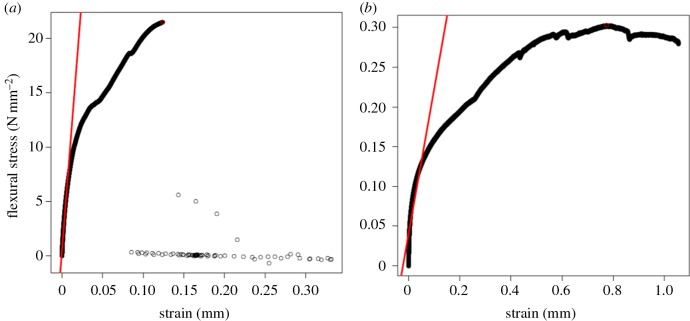
Mechanical testing results of dried (*a*) and hydrated baleen samples (*b*) display similar curves but with quite different scales. This representative sample of dried baleen failed fractured into many pieces at 21.5 N mm^−2^, whereas this typical wet baleen sample did not fail but withstood only one-tenth as much stress (0.3 N mm^−2^) before it was bent so much that the materials testing machine could not bend it any further—it reached the limit that the compressive piston could travel. The largest slope of the stress–strain curves (straight red line) was measured as flexural stiffness.

**Figure 4.**
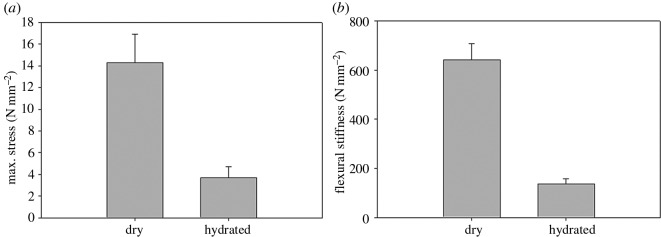
Maximum recorded flexural stress forces were 4× higher in dried versus wet baleen samples (mean 14.29 versus 3.69 N mm^−2^) and calculated flexural stiffness was almost 11× higher in dried versus wet baleen samples (mean 633.09 versus 58.16 N mm^−2^, shown ±1 s.d.).

Results of the uniaxial bending testing (figures 3 and 4) confirm that baleen is strong. Some air-dried samples withstood compressive forces of more than 1 kN before yielding and failing; some wet samples withstood similarly high forces (approx. 1 kN) before returning to their original shape, thus demonstrating elasticity. These findings show that hydrated baleen is also exceedingly flexible, especially where it is thinnest (less than 3 mm). However, in most trials the dried baleen exhibited a yield point (i.e. the stress at which the baleen began to deform plastically, signified by a change in the slope of the stress–strain curve) in the range of 10–15 N mm^−2^ (10–15 MPa) and a failure point (i.e. snapping or cracking, often into multiple pieces) at around 20–30 N mm^−2^ (20–30 MPa). Figure 3 shows typical stress–strain curves for dried and hydrated baleen. The loading curve of wet baleen has a shape similar to that of dried baleen except with different scaling on the *y*-axis, for it shows great physical deformation (strain) when much lower forces are applied (figure 1). Wet baleen samples yielded at 0.1–0.2 N mm^−2^ and maximum stresses of 0.3–0.4 N mm^−2^. Because the wet samples never failed (broke), the maximum stresses recorded in these trials were limited by the dimensions of the testing devices. The mean maximum recorded stress was significantly (*p* = 0.02) higher in dried (14.29 N mm^−2^) versus hydrated (3.69 N mm^−2^) baleen. The flexural stiffness was more than 10× higher in dried (633.09N mm^−2^) versus hydrated (58.16 N mm^−2^) baleen samples (figure 4); *t*-testing confirmed that this is also statistically significant (*p* = 0.01). Statistical analysis by *t*-test also showed that results from the Instron and Mark-10 universal testing machines were not significantly different (*p* = 0.82).

Mechanical testing was also performed on control materials to determine if wet versus dry samples showed differences in stress, strain or flexural stiffness. *t*-tests were used to compare results statistically. No differences were found in any material properties for wet versus dry aluminium (*n* = 5, *p* = 0.93) or HDPE plastic (*n* = 5, *p* = 0.68), although the wet and dry samples of lauan plywood did exhibit differences in maximum recorded stress (*n* = 5, mean = 28 N mm^−2^ dry with s.d. = 4.33 and mean = 16 N mm^−2^ wet with s.d. = 7.24; *p* = 0.31) and flexural stiffness (*n* = 3, mean = 368 N mm^−2^ dry with s.d. = 24.93 and mean = 252 N mm^−2^ wet with s.d. = 18.81; *p* = 0.27).

**Figure 5.**
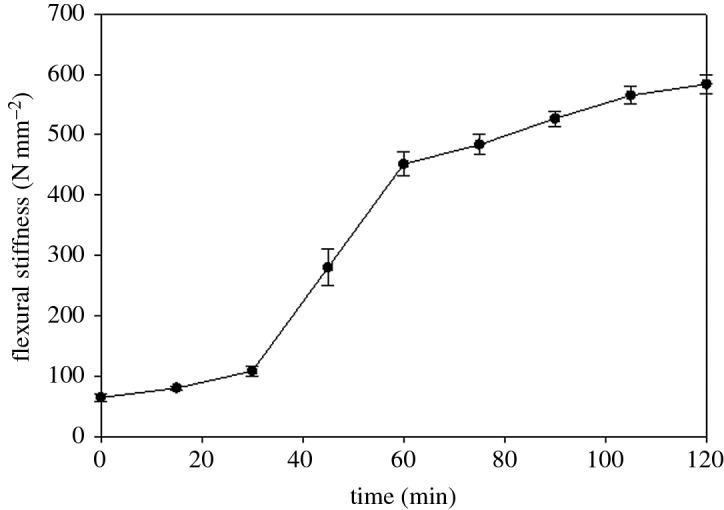
Measurements of flexural stiffness at 15 min intervals after removal from water show rapid drying and consequent change in baleen's mechanical properties, with greatest change occurring between 30 and 60 min post-submergence. Boxes indicate 25th–75th percentile with line marking mean data; error bar whiskers show 10th to 90th percentile of data.

The baleen samples that were tested at 15 min intervals after removal from water, then placed in air at 22°C with approximately 30% humidity, demonstrated marked and rapid changes in mechanical stiffness (figure 5) that were especially notable within 30–60 min after removal from water. During this half hour period the baleen samples (*n* = 30) showed over a fourfold increase in mean flexural stiffness, from 108.4 to 451.3 N mm^−2^. This experiment was terminated after 2 h because it seemed the change in mechanical properties had halted, but the data show (figure 5) that stiffness continued to increase slightly as the tissue continued to dry.

## 4. Discussion

The impact of hydration on alpha-keratin in mammals [27,35–38], as well as on invertebrate keratins which include β-pleated sheets [39], has been previously documented, although Feughelman [40] claimed that keratin fibres are mostly impervious to hydration. Given its supposed waterproofing or water resistance abilities (similar to chitin) and its integumentary ubiquity across many animal taxa, keratin frequently comes into contact with water. Our findings conclusively demonstrate that when re-submerged, dried mysticete baleen absorbs substantial amounts of water (we found that wet baleen is 32–34% water by weight depending on species) and that as its water content increases the surface of baleen becomes notably hydrophilic.

**Figure 6.**
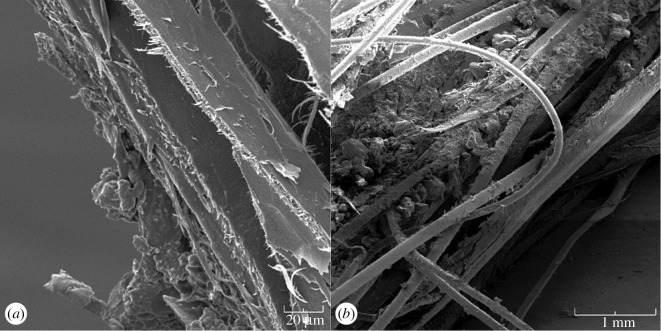
Scanning electron micrographs (taken by Todd Sformo at the University of Alaska Fairbanks, Advanced Instrumentation Laboratory) show baleen tissue's ‘grainy’, anisotropic nature owing to separation of cortical keratin layers at the plate edge (*a*, 600×) as well as emergence of underlying keratin horn tubes to become exposed baleen fringes (*b*, 25×). The rough surfaces and exposed spaces presumably aid in water penetration and adhesion.

The mechanism behind this simple change has been investigated, most often via investigation of wool or bovid horn fibres [23,26]. Alpha-keratin is sometimes deemed to be a waterproof protein, but its helical fibres expand and uncoil when exposed to water, making the protein less stiff [8,27]. Although baleen is exclusively keratinous, it is possible that water seeps into tiny surface cracks and adheres to keratin between the medullary horn tubes and interstitial keratin (figure 6) as well as coheres to other water molecules. Leeder & Watt [23] distinguished bound from condensed (mobile) water on keratin; they concluded that at lower (less than or equal to 30%) humidity, 1–3 water molecules attach to keratin's individual amino groups and side chains, and at high (greater than or equal to 80%) humidity additional water is added not by direct binding to the protein itself but via attraction to adjacent water molecules already present. Leeder & Watt [23] also found that at high levels of humidity (greater than or equal to 80%), keratin swells and undergoes a conformational shift via torsional expansion, exposing additional ‘hydrophilic sorption sites’ that increase the extent of hydration. Maeda [26] summarized previous studies of keratin in water and investigated parameters of keratin hydration at temperatures from −160° to 150°C, concluding that at extremely low (less than or equal to −70°C) or high (greater than or equal to 50°C) temperatures, tightly bound water stiffens keratin, whereas at more moderate temperatures, water molecules loosely bound to polar sites on polypeptide side chains form ‘water-bridges’ via hydrogen bonding, thereby increasing keratin's elasticity. Importantly, the moderate temperatures of Maeda's [26] study and related reports on keratin hydration [22,24,41] reflect the normal range of ambient water temperatures (approx. 0–25°C) encountered by all mysticete whale species in their natural habitats.

Hydrated, softened keratin proteins will exhibit greater surface area to which water can further adhere. The square samples used in this study, having unnaturally sawn edges, may thus have allowed entry of water. Nonetheless, further experiments with three whole, small (mean 56 cm long) plates of right whale baleen and four whole, small (mean 32.1 cm long) plates of bowhead baleen possessing only natural edges (except for where they were removed from the palate along the dorsal limit) also showed a mean increase in weight from hydration of 28.6% (*n* = 3, $\bar{x}=5.63\;\text{g}$ gained, s.d. = 3.61) and 34.3% (*n* = 4, $\bar{x}=7.31\;\text{g}$ gained, s.d. = 2.42), respectively, indicating that baleen in its natural form and condition (i.e. with a single artificially cut edge) likewise absorbs substantial amounts of water.

The overall finding that baleen's properties are strongly affected by hydration may not be surprising given the familiar softening effects of submergence in water on fingernails, another hard mammalian tissue comprised solely of alpha-keratin [10,11]. In addition, native Inupiat-Inuit artists who fashion strips or sections of baleen into baskets, crafts, and decorative artwork begin by rehydrating baleen over a period of days or weeks to make it pliable [42]. However, even if our findings are somewhat predictable, they are nonetheless important for several notable reasons.

First, our findings clearly document the powerful effects of hydration on biological tissues composed of keratin. Second, the dramatic scope of the mechanical and physical changes produced by baleen's hydration was unforeseeable and much greater than anticipated. Third, these results suggest that baleen's development, emergence and use during filter feeding occur with a material that, although strong, is much more pliant and malleable than commonly supposed (except by native artists who have been rehydrating baleen for centuries). This, in turn, suggests that baleen, owing to its eruption and use in an exclusively aqueous environment, is unlike other mammalian epidermal alpha-keratins that are formed and used in air. As Szewciw *et al.* [13] explained, baleen is different from other mammalian keratins in that it is never air-dried (*in vivo*) during any stage of development, most notably as it forms and emerges from the gums [43–45], but also during any later portion of life history. Szewciw *et al*. [13] reported high levels of calcium salts in baleen samples from minke (*Balaenoptera acutorostrata*), sei (*Balaenoptera borealis*) and humpback (*Megaptera novaeangliae*) whales, and concluded that impregnation with minerals, particularly via calcification, provides crucial mechanical reinforcement to stiffen baleen tissue in the absence of normal air-drying. Whales obviously spend their entire life cycle underwater. Aside from brief moments when a whale opens its mouth at the water's surface, baleen is never at any point exposed to air, and even then very humid air. This poses a challenge for a tissue that must ‘cure’ to harden into a strong, tough material. Nails, claws, hairs and other hard, fibre-reinforced alpha-keratin tissues develop in dry air, even in marine mammals, most of which (unlike cetaceans) spend considerable time hauled out on land. Pautard [46] suggested baleen's elevated calcium levels also resist abrasive fraying. Such calcification is absent in other mammalian alpha-keratinous tissues.

All baleen specimens in curated museum collections are stored or displayed in air, and these are generally the specimens that are sampled for investigations of baleen's structure and function. Studies that use baleen taken directly from naturally deceased or hunted animals invariably involve tissue that has been exposed to air for hours if not days or much longer. This undoubtedly alters baleen's physical and mechanical properties given that it has never previously been exposed to air. Our results clearly show that even when dried, baleen is a remarkably strong tissue, routinely absorbing yield stresses of 50 MPa or more before fracturing in compression tests. Upon failure, the dried tissue samples splintered into dozens of sharp shards that were scattered up to 5 m across the testing room. By contrast, the wet samples never reached a failure point; instead they bent easily and extruded small puddles of water from the point of contact where the compressive piston was applied (figure 1*b*). After testing, the wet samples immediately recovered their original positions, with no externally visible changes and with loading profiles demonstrating an apparent elastic hysteresis loop.

Our findings are comparable with previously published data on baleen's material properties. St Aubin *et al*. [31] reported that ‘the breaking strength of baleen plates from fin and grey whales was comparable with that of buffalo horn,’ in the range of 2–9 MPa. They also reported that ‘the stiffness of baleen was somewhat less than that of other keratinized tissues’, but without quantifying this difference. Our samples of dried baleen failed at 20–30 MPa, whereas our hydrated baleen samples ‘maxed out’ at 0.3–0.4 MPa. The values that St. Aubin *et al*. [31] documented fit neatly into the range we found between dried and hydrated baleen, yet the previous investigators did not convey the great difference between dry and wet baleen, nor did their understatement concerning baleen's stiffness take into account the major role that hydration plays.

Baleen used in this study was cut into sections with a band saw, but it was difficult to section the baleen with a powered or hand saw or with other cutting tools (e.g. tissue shears or snips) because dried baleen was brittle and would easily snap when sawed. By contrast, fully hydrated baleen was so flexible and ductile that it was equally challenging to cut with a saw. Further complicating the cutting process was the anisotropic nature of baleen material, with a ‘grain’ (similar to wood) produced by the core of cylindrical keratinous horn tubes (figure 6). When loaded, baleen almost invariably (84 times out of 87 recorded instances, or 97%) fails lengthwise, splitting between these tubes rather than across the grain they form. The role of anisotropy in resisting crack propagation in mammalian keratin has been explored [47]. In life, such failure would merely promote the erosive wear process that reveals the horn tubes [14], which when exposed form the crucial fringes that perform most of the work of filtration for feeding, especially in balaenid (right and bowhead) whales [18].

The curved ‘trailing’ (lateral or labial) edge of baleen plates, which gives them a hydrofoil camber, was excluded from most square samples used for strength testing, as a flat square was deemed desirable. However, tissue sections that included this curved edge showed increased strength and resistance to bending. The sample size was not large enough to quantify this effect; it demands further investigation.

The results of this study are additionally noteworthy for three more general reasons.

(i) Many biologists (and non-scientists) have seen or touched samples of baleen at museums or via whale watch excursions, and the perception of baleen is—like virtually all published accounts including those in anatomical or marine mammal literature [7]—based on the mistaken notion that baleen is a stiff, rigid material. Our results conclusively demonstrate that this popular impression is inaccurate. Baleen *in vivo* (or otherwise hydrated) is extraordinarily flexible and ductile: more like a soft plastic than a hard plastic. In short, dried baleen is familiar to many biologists, but it is biologically unrealistic.
(ii) Consequently, our overall understanding of normal baleen function is also likely to be deficient, especially with regard to the physical, small-scale mechanisms of filtration, such as whether baleen filtration principally involves direct interception or inertial impaction of particles [48], and whether water and water-borne prey items chiefly flow through or along the filter [18], etc. Baleen may function less as a passive sieve and more as a dynamic filter that traps accumulated prey by governing intraoral flow fields [18]. Most general accounts of baleen's filtration action [21,49–52] stem from the conventional perception of baleen as at best a predominantly rigid and somewhat flexible material, not as an exceptionally flexible material. Even flow tank experiments that have used fully submerged and thus fully hydrated baleen specimens [17,17,20] have nonetheless relied on baleen that has for at least some *post mortem* period been allowed to dry in air, usually for days, perhaps irreversibly altering its material and physical properties. Our results confirm that material or functional studies of baleen, such as mechanical testing or laboratory experiments, should use fully hydrated baleen samples or at least acknowledge hydration's importance and take it into account during analysis. Future functional studies should as much as possible use fresh baleen that is immediately and continuously stored in water. This has major implications for the study of mysticete feeding morphology and biomechanics as well as the energetics of filtration and drag and, as this relates to diet and potential oral entanglement, ecology and conservation.
(iii) Baleen's high levels of hydration probably affect its interaction with non-polar, hydrophobic water-borne substances, especially oil from anthropogenic point sources (such as ship or oil well spills) as well as natural sources, given that oil droplets are a major component of copepods and other prime constituents of mysticete diets. The potential role of prey-borne oil in affecting filtration has been briefly speculated upon [17], as has the potential for crude and refined oil to ‘stick’ to baleen, possibly clogging the oral filter and/or becoming ingested [31,53,54]. Now that hydrated baleen's hydrophilic—and potentially oleophobic—nature is more clearly understood, additional studies must investigate the interaction of baleen with oils and related petroleum hydrocarbons as well as other non-polar toxins, contaminants and chemical pollutants that whales might regularly encounter and potentially ingest in their natural environment, with major implications for the study of cetacean health and toxicology.

Work is ongoing to broaden the results of this study, involving comparative analysis of baleen tissue from whales of diverse species and age classes. Preliminary results of hydration and mechanical testing from whales of four other species (bowhead, *Balaena mysticetus*; blue, *Balaenoptera musculus*; fin, *Balaenoptera physalus*; humpback, *Megaptera novaeangliae*) indicate levels and effects of hydration similar to those reported here in *Eubalaena*, but less flexibility (except in the case of *Balaena*). Other comparisons to be tested or analysed include potential differences in baleen plates from different positions along a rack (e.g. from cranial-most to caudal-most plates) as well as along the dorsoventral length of individual plates, from the oldest baleen at the ventral-most vertex of a plate to the newest baleen emerging from the dorsal gingival tissue.

## Supplementary Material

Supplemental File 1 converted original test data Supplemental File 2 computed stress strain data Supplemental File 3 data plots

## Data accessibility

Datasets are available in the electronic supplementary material.
